# Revisiting two thousand hinge fractures in open wedge high tibial osteotomy with a fifty years review: the oscillating saw cannot replace the traditional “ear-hand” dialogue between osteotome and hammer to estimate the elastic modulus of bone

**DOI:** 10.1051/sicotj/2024060

**Published:** 2025-01-20

**Authors:** Claire Bastard, Guillaume Haiat, Philippe Hernigou

**Affiliations:** 1 Hôpital Saint-Antoine 184 Rue du Faubourg Saint-Antoine 75012 Paris France; 2 CNRS, Université Gustave Eiffel, University Paris East (UPEC) 5 Bd Descartes 77420 Champs-sur-Marne France; 3 Hospital Henri Mondor, University Paris East (UPEC) Avenue du Marechal de Lattre de Tassigny 94000 Creteil France

**Keywords:** High tibial osteotomy, Open wedge, Opening wedge, Hinge fracture, Osteotome, Oscillating saw, Impact-based analysis method, Instrumented hammer, Piezoelectric force sensor

## Abstract

*Background*: Hinge fracture on the lateral part of the tibia (LHF) is a common complication of medial Open Wedge High Tibial Osteotomy (OWHTO). Many factors have been described as risks for these fractures, but no study has compared an osteotome or an oscillating saw to prevent LHF following OWHTO. *Methods*: This “propensity-score-matched” (PSM) study was conducted from data obtained in the literature from 1974 to November 2024. A total of 10,368 knees with OWHTO were identified. After 1:1 matching based on correction amount, posterior slope change, surgeon’s experience, the osteotome and oscillating groups comprised 2760 knees each. *Results*: Among the 5520 knees of the PSM population, the prevalence of LHF was 6.1% in the osteotome alone group (168 cases), and 22% in the oscillating saw group (607 cases). The osteotome group had a significant lower prevalence of hinge fracture than the oscillating saw group (OR, 0.23; 95% CI, 0.19 to 0.27; *p* < 0.0001) and a lower rate of clinically relevant hinge fractures with revision (OR, 0.34; 95% CI, 0.25 to 0.45; *p* < 0.001. *Discussion*: The osteotome may be an appropriate method for preventing hinge fractures following OWHTO.

## Introduction

Opening wedge tibial osteotomy was pioneered over 50 years ago as an alternative for knee alignment correction in patients with medial compartment osteoarthritis, with the first publication in the English literature of this technique [1]. In this first report analyzing OWHTO performed since 1974 (50 years ago), Hernigou et al. [1] were also the first to report a lateral cortex fracture complicating this technique. This complication resulted in varus displacement of the osteotomy before osteotomy healing in some patients (12%) in the absence of osteosynthesis. Since this is a first report, the senior author had introduced osteosynthesis [2] as early in 1987, and full weight bearing with locked screws even in simultaneous bilateral tibial osteotomy [3], with a decrease of hinge fractures to 2% in his practice. After 50 years, among the 100 most-cited papers about high tibial osteotomy published from 1970 to 2023, the study [1] (“Proximal tibial osteotomy for osteoarthritis with varus deformity – a 10–13-year follow-up study”) remains “number 1” for citations [4]. While this technique offers considerable advantages when compared to closing wedge technique [5, 6], a lateral fracture risk in opening technique is highly related to nonunion [7, 8].

Until now, some recent systematic reviews have been performed to summarize the risk factors and the frequency of this complication [9–11]. However, most authors have never mentioned an essential element as a risk factor: using the oscillating saw in the first part of the osteotomy instead of performing it only with an osteotome as in the original technique. Considering that using an oscillating saw can increase the risk of hinge fracture, we investigated this factor in a systematic review of OWHTO.

## Materials and methods

### Study design

It was retrospective and performed with data of literature. In total, 10,368 knees with OWHTO were identified. Informed consent was not required. A “propensity-score-matched” (PSM) cohort study of patients was conducted among these 10,368 knees with OWHTO reported in literature. This study followed the “Strengthening the Reporting of Observational Studies in Epidemiology” reporting guidelines.

Due to the absence of guidelines or expert consensus on the perioperative use of osteotome or oscillating saw for patients undergoing OWHTO, most centers lack specific criteria for selecting these tools in routine clinical practice. Consequently, physicians rely on their clinical experience to choose the tools and methods they use. Three methods are used in the literature: 1) the use of only osteotome, 2) the oscillating saw, or 3) association of oscillating saw and osteotome.

### Literature search

This systematic review was conducted following Cochrane Review Methods, using “PRISMA guidelines” [12] to identify and extract relevant articles. A structured search was performed across different databases: Cochrane Library, PubMed (MEDLINE), EMBASE. Studies published from 1986 up to October 2024 were analyzed.

The search query used the key words “high tibial osteotomy,” “proximal tibial osteotomy,” “valgus knee osteotomy,” “lateral hinge fracture,” “plateau tibial fracture,” “lateral cortex fracture,” and “opposite cortical fracture.”

### Study selection

The titles and abstracts were screened with artificial intelligence [13] using natural language processing, which allowed the interpretation of human language in selecting papers. The criteria for study selection were: patients treated with medial OWHTO for cartilage defects and osteoarthritis, for anterior cruciate ligament deficiency, or symptomatic knee malalignment, osteonecrosis, osteotomy associated to anterior cruciate repair, associated to TKA or UKA, iterative osteotomy; studies reporting medial OWHTO whatever technique, the use of only osteotome, or only oscillating saw, or association of oscillating saw and osteotome; studies with detection of lateral part of the tibia (LHF) with fluoroscopy, radiographs or computed tomography (CT) scan. For eligibility two reviewers (CB and PH) independently evaluated the titles and abstracts of the identified studies. Discrepancy was discussed with the third author. Only full text articles (without language restriction) were included in this analysis.

### Data extraction

Specific data regarding surgical technique included correction target, type of OWHTO, fixation method, gap filling, and postoperative rehabilitation. The incidence of LHF was detected by intraoperative fluoroscopy or immediate plain radiography and additional CT. Takeuchi et al. [7] type (I, II, and III), and detailed management of the LHF, such as additional fixation or changing rehabilitation protocol, was recorded. Based on Takeuchi et al. stable (I) and unstable (II, III) LHF were assigned to subgroups. Radiologic outcomes included the following parameters: 1) correction amount: opening gap distance (measured by intraoperatively measurement or immediate postoperative plain radiographs or CT), correction angle (immediate postoperative measurement – preoperative measurement) of hip-knee-ankle angle and medial proximal tibial angle; 2) correction loss (final follow-up measurement – immediate post-operative measurement) of the HKA angle; 3) posterior slope change.

The specific data related to the surgical technique included the correction, the type of OWHTO, the fixation method, the gap filling, and the postoperative rehabilitation protocol. The incidence of LHF was identified through intraoperative fluoroscopy, immediate plain radiography, or additional CT scans. The classification of LHF followed the Takeuchi et al. [7] system (Types I, II, and III), with detailed management recorded, such as additional fixation or modifications to the rehabilitation protocol. Based on Takeuchi’s classification, LHFs were categorized into stable (Type I) and unstable (Types II and III) subgroups. Radiologic outcomes were assessed using the following parameters: correction amount, the opening gap distance (measured intraoperatively or via immediate postoperative radiographs), and changes in the hip-knee-ankle (HKA) angle or medial proximal tibial angle (postoperative vs. preoperative measurements); correction loss, defined as the difference between the final follow-up and immediate postoperative HKA angle measurements; and changes in the posterior slope.

#### Bias

Authors or journals are more likely to publish research with positive results. Here the analysis is centered on a complication (hinge fracture) and this may be a cause of publication bias. In some of the studies we used individual patient data, allowing greater flexibility for the analysis and issues not covered in the published trials. This could be done when the author of their article was present in the published series. However, obtaining the original patient data from the trials can be challenging. The risk of bias was classified as high, low, or unclear.

### Definition of outcomes and baseline covariates

The primary outcome was LHF. The second outcome was to evaluate the severity of LHF. A clinically “important” LHF was defined associating system described by Takeuchi et al. [7] and management of the LHF such as a need for an additional fixation.

To avoid confounding effects from baseline covariates, a PSM analysis was performed on the basis of baseline covariates. This method [14] enables investigators to measure the effect of an intervention without necessitating the randomization of subjects to that intervention. The following baseline covariates data were collected on the basis of articles, published in the last decades, that have explored the features associated with LHF: correction amount [15–20]; 1) opening gap distance [21–27] (measured by intraoperatively measurement or immediate postoperative plain radiographs or CT), correction angle (immediate postoperative measurement – preoperative measurement) of hip-knee-ankle (HKA) angle and medial proximal tibial angle (MPTA); 2) correction loss (final follow-up measurement – immediate post-operative measurement) of the HKA angle and MPTA; 3) posterior slope change; and 4) time until radiologic bone union; 5) the experience of the surgeon with the number of osteotomies reported (>100 osteotomies as experience).

To minimize confounding effects from baseline covariates, a PSM analysis was conducted based on these variables. This approach [14] allows researchers to assess the impact of an intervention without requiring subject randomization. Baseline covariate data were collected according to studies published in recent decades that have examined characteristics associated with LHF. These covariates included: correction amount [15–20]; open gap distance [21–27]; correction angle between the immediate postoperative and preoperative measurements; correction loss in frontal and sagittal planes.

### Statistical analysis

Descriptive statistics were calculated, presenting categorical variables as counts and frequencies, while continuous variables were expressed as mean and standard deviation (SD). Propensity scores for the use of an oscillating saw versus an osteotome were estimated through multiple logistic regression, adjusting for baseline covariates. A “standardized” mean difference (SMD) threshold of 0.10 was used to evaluate covariate balance between the osteotome and non-osteotome groups in both the overall population and the PSM cohort. Crude and adjusted odd ratios (ORs) for the occurrence of lateral hinge fractures were determined using univariate and multiple logistic regression analyses.

## Results

### Identification of studies

**Figure 1:**
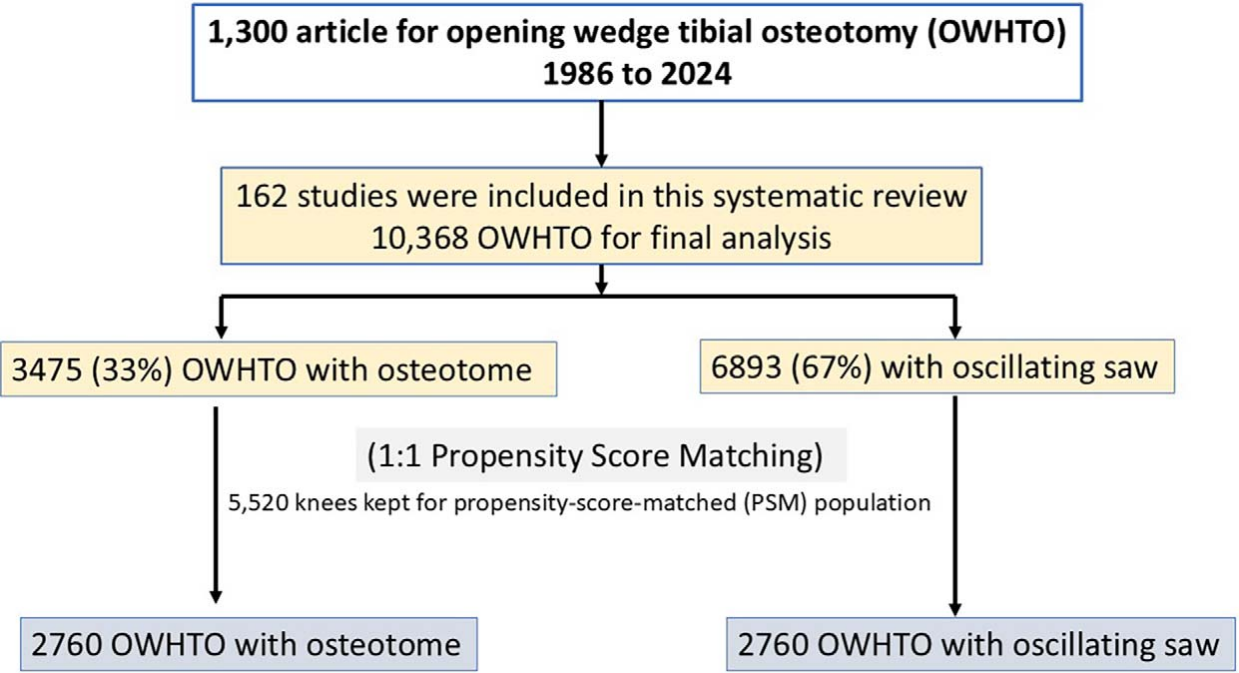
Study flow and patient selection: Exclusion were animal or cadaveric studies, biomechanical and editorial articles, editorials. Review papers, case reports, conference abstracts were also excluded.

1300 results were obtained for “opening wedge tibial osteotomy.” After reading the title, 982 studies were kept. After the removal of 748 studies (cadavers, animals or biomechanic, editorials, reviews, case reports, abstracts), 234 remained; of them, 72 were excluded after review of the full text. Therefore, 162 studies were the relevant part of this systematic review. The flow diagram and inclusion is shown in Figure 1.

### Incidence of lateral hinge fractures (LHF) in the global series

**Figure 2:**
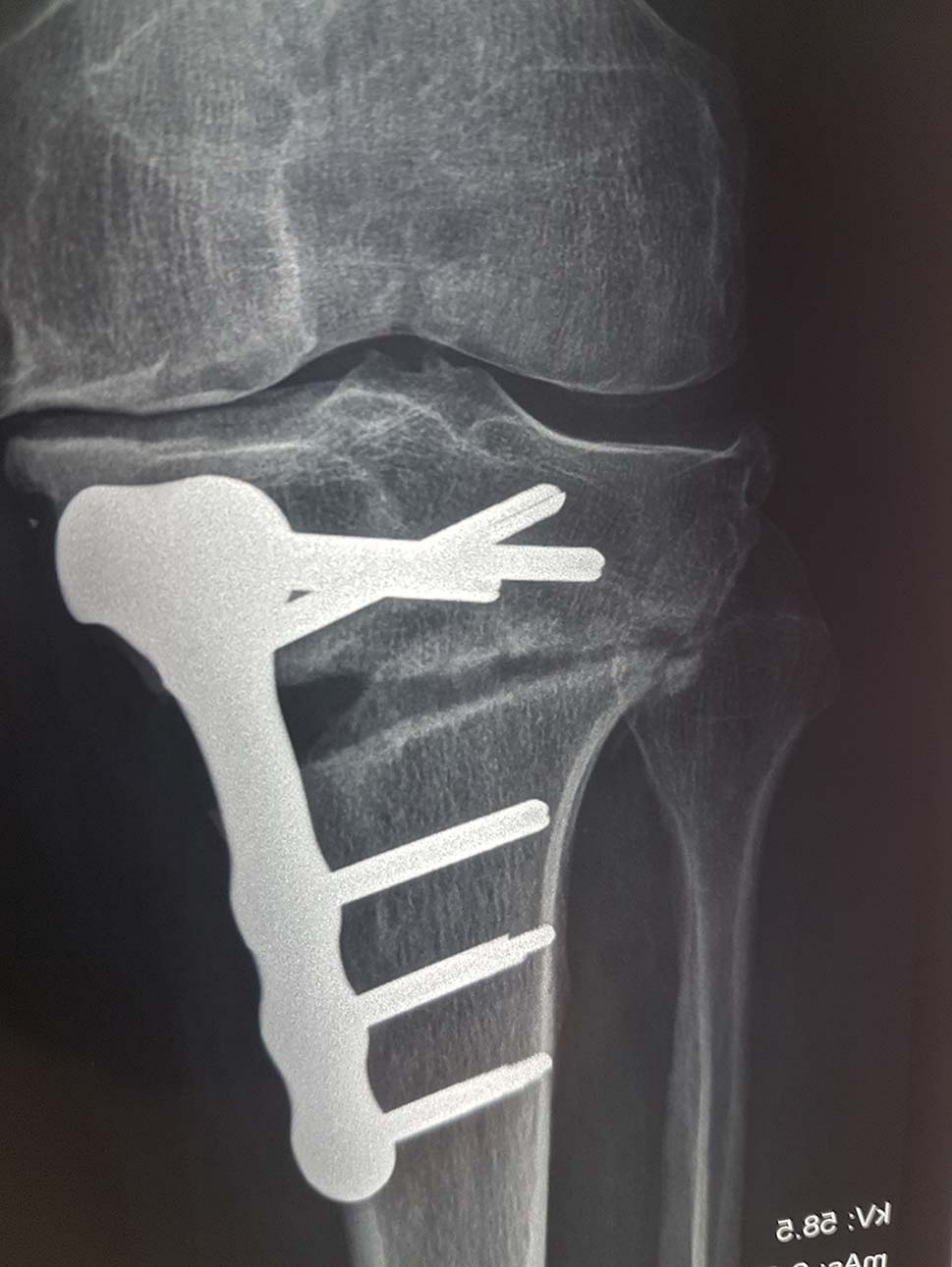
Lateral hinge fracture.

The average number of knees with OWHTO was 64 knees per article with 10,368 OWHTO, and the estimate of hinge fracture (Figure 2) in the pooled studies was 19.4% (range, 5.2% to 32.5%;), with 2014 as a total number of LHF announced in the total articles. Of the 162 studies, 133 reported osteotomies for the treatment of osteoarthritis, 11 as associated treatment for ligamentoplasty, 5 for knee osteonecrosis, 4 as associated treatment for regenerative cartilage defect; 4 studies were about osteotomies associated with TKA or UKA, 3 were performed for tibial plateau fracture sequelae, and 2 were iterative osteotomy.

The use of the “osteotome” alone without the oscillating saw was mentioned in 34 articles. Most of these papers are in French where the osteotomy was popularized and these papers are cited independently in Table 1 to avoid to increase the number of references of this article. The other papers mentioned association of oscillating saw and osteotome (Table 2).

**Table 1:** Papers with osteotomes.

Author	Journal	Year	DOI
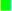 Hernigou P	J Bone Joint Surg Am	1987	https://doi.org/10.2106/00004623-198769030-00005
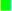 Hernigou P	Rev Chir Orthop	1988	74(3), 232–237
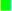 Hernigou P	Int Orthop	2020	https://doi.org/10.1007/s00264-019-04385-z
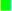 Hernigou P	Rev Chir Orthop	1996	82, 241–250
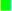 Jenny J	Rev Chir Orthop	1998	84, 350–357
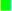 Segal P	Rev Chir Orthop	1992	78(Suppl I), 88–94
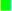 Bonnevialle P	Rev Chir Orthop	2002	88, 486–492
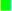 Goutallier D	Rev Chir Orthop	1992	78, 138–144
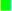 Bove JC	Rev Chir Orthop	2002	88, 480–485
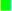 Gacon G	Eur J Surg Traumatol	1997	7, 130
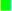 Lascar T	Ann Orthop Ouest	1996	98, 48–49
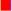 Romanet JP	Rev Chir Orthop	1996	87(supp II), 17
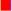 Lavalle F	Revue de chirurgie	2004	21–28
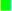 Dehoux, E.	Rev Chir Orthop	2005	https://doi.org/10.1016/s0035-1040(05)84292-8
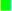 Villatte G	Rev Chir Orthop	2015	https://doi.org/10.1016/j.rcot.2015.10.008
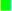 Dubrana F	Rev Chir Orthop	2008	94, S2–S21
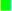 Brosset T	OTSR	2011	97, 705–11
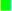 Hernigou P	Int Orthop	2015	39, 1295–300
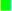 Saragaglia D	Int Orthop	2011	35, 1151–1156
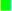 Hernigou P	Int Orthop	2010	34, 191–199
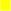 Schallberger	KSSTA	2011	19, 122–127
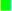 Amzallag J	KSSTA	2013	21, 255–259
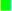 Gouin F	OTSR	2010	96, 637–645
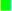 Hernigou P	Int Orthop	2013	https://doi.org/10.1007/s00264-013-2066-3
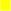 Ducat A	OTSR	2012	https://doi.org/10.1016/j.otsr.2011.08.013
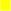 Siboni R	OTSR	2018	104(4), 473–476
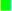 Hernigou P	Knee	2001	https://doi.org/10.1016/s0968-0160(00)00061-2
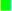 Hernigou P	Int Orthop	2015	https://doi.org/10.1007/s00264-014-2633-2

	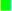 Low	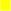 Unclear	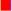 High
	Risk of bias		

**Table 2:** Papers with oscillating saw.

Author	Journal	Year	DOI
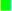 Yoo MJ	KSRR	2016	https://doi.org/10.5792/ksrr.15.075
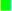 Takeuchi R	J Arthro	2008	https://doi.org/10.1016/j.arthro.2008.08.015
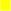 Yabuuchi K	OJSM	2020	https://doi.org/10.1177/2325967120922535
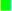 Han SB	J Arthro	2019	https://doi.org/10.1016/j.arth.2019.01.026
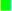 Lee BS	AJSM	2019	https://doi.org/10.1177/0363546519836949
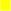 Meidinger G	KSSTA	2011	https://doi.org/10.1007/s00167-010-1335-6
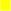 Martin R	AJSM	2014	https://doi.org/10.1177/0363546514525929
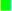 Nakamura R	BJJ	2015	https://doi.org/10.1302/0301-620X.97B9.34949
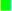 Dexel J	J Arthro	2017	https://doi.org/10.1007/s00167-015-3730-5
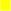 Kim TW	J Arthro	2019	https://doi.org/10.1016/j.arthro.2019.01.044
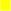 van Raaij TM	Act Orthop	2008	https://doi.org/10.1080/17453670710015508
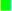 Schröter S	J Arthro	2015	https://doi.org/10.1016/j.arthro.2014.08.028
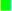 Song KY	AOTS	2020	https://doi.org/10.1007/s00402-019-03237-0
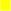 Goshima K	KSSTA	2019	https://doi.org/10.1007/s00167-018-5334-3
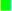 Kim KI	J Arthro	2018	https://doi.org/10.1016/j.arthro.2018.07.022
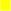 Devgan A	Med J Malaysia	2003	58(1), 62–68
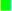 Chen YN	JOSR	2020	https://doi.org/10.1186/s13018-020-01922-0
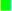 Dessyn E	J Arthro	2020	https://doi.org/10.1007/s00167-019-05404-7
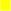 Gulagaci F	KSSTA	2020	https://doi.org/10.1007/s00167-019-05806-7
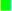 Kim TW	J Arthro	2019	https://doi.org/10.1016/j.arthro.2019.01.044
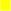 Staubli AE	Injury	2003	https://doi.org/10.1016/j.injury.2003.09.025
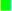 Koshino T	JBJS Am	2003	https://doi.org/10.2106/00004623-200301000-00013
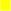 Spahn G.	AOTS	2004	https://doi.org/10.1007/s00402-003-0588-7
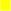 Amendola A	J K Surg	2004	https://doi.org/10.1055/s-0030-1248216
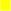 Warden SJ	KSSTA	2005	https://doi.org/10.1007/s00167-003-0485-1
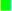 Xiangzhi Y	J Arthro	2024	https://doi.org/10.1016/j.arth.2024.11.003
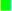 Hung YT	Orthop J Sports Med	2024	https://doi.org/10.1177/23259671241277827
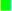 Guo H	J Orthop Surg Res	2024	https://doi.org/10.1186/s13018-024-04909-3
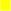 Soykan B	J Exp Orthop	2024	https://doi.org/10.1002/jeo2.12086
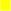 Otsuki S	J Exp Orthop	2023	https://doi.org/10.1186/s40634-023-00701-0
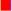 Li J	Sci Rep	2023	https://doi.org/10.1038/s41598-023-44051-4
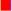 Schröter S	Z Orthop Unfall	2024	https://doi.org/10.1055/a-2120-0993
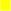 Yoshida K	KSSTA	2023	https://doi.org/10.1007/s00167-023-07544-3
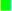 Kim SM	Arthroscopy	2024	https://doi.org/10.1016/j.arthro.2023.07.054

	Risk of bias		
	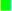 Low	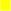 Unclear	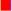 High

The estimate of hinge fracture was 5.7% (95% CI, 3.2% to 11.1%) with OWHTO performed with osteotome alone without oscillating saw; incidence of LHF was 25.1% (95% CI, 11.2% to 37.1%) with oscillating saw alone and 18.4% (95% CI, 12.5% to 36.1%) when the association of both.

### Study Population for propensity-score-matched analysis and baseline covariates

In total, 10,368 knees (9746 patients) that underwent OWHTO surgical treatment were identified: 3475 (33%) when the osteotome was used alone (osteotome group), and 6893 (67%) in the group with oscillating saw technique. The mean age was 59 ± 26 years, and 5942 patients (56%) were men.

In order to compare the two groups, 2760 pairs of individuals (1:1 match) were obtained after PSM analysis, including 2760 knees with the oscillating saw and 2760 knees with the osteotome (Figure 1). Standardized mean differences (SMDs) assessed the balance of baseline covariates. SMD values were <0.10, indicating few differences between groups (Table 3) after matching.

**Table 3:** Propensity score matching.

	Overall	Osteotome	Oscillating saw	
Variable	*N* = 5520	*N* = 2760	*N* = 2760	SMD
Male (Nb)	2633	1343	1290	0.02
Age (Mean, years)	65	66	64	0.05
Indication (Nb)				
Osteoarthritis	3876	1893	1983	0.03
Cartilage (sport)	640	303	337	0.05
ACL ligament	531	261	270	0.06
Osteonecrosis	275	142	133	0.03
With TKA or UKA	123	64	59	0.07
Iterative HTO	75	42	33	0.09
Gap size (Nb)				
≥10 mm	1954	982	972	0.04
<10 mm	3566	1778	1798	0.04
Correction (Nb)				
≥10°	1837	892	935	0.03
<10°	3683	1868	1825	0.02
Posterior slope				
Increase	1443	821	622	0.08
Decrease	4087	1939	2138	0.07

SMD: standardized mean difference.

### Association of osteotome use with lateral hinge fracture prevalence

Among the PSM population (5520 knees), the prevalence of LHF was 6.1% in the osteotome alone group (168 cases among 2760 knees) and 22% in the oscillating saw group (607 cases). On regression analysis, the group with osteotome had a lower LHF prevalence than the group with oscillating saw (OR, 0.23; 95% CI, 0.19 to 0.27; *p* < 0.0001).

The prevalence of clinically “important” LHF was 2.07% (56 cases among 2760 knees) in the osteotome group and 5.81% (160 cases) in the oscillating saw group. The osteotome group had a lower prevalence of clinically important hinge fracture than the oscillating saw group (OR, 0.34; 95% CI, 0.25 to 0.45; *p* < 0.001).

### Subgroup and interaction analyses of the effect of osteotome use on lateral hinge fracture prevalence

**Figure 3:**
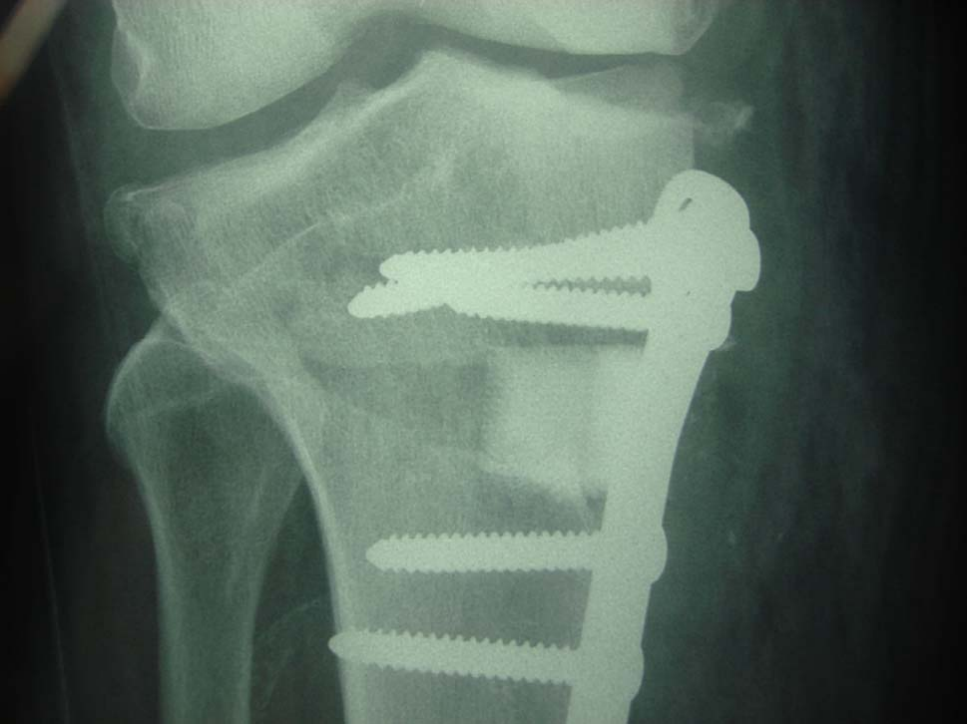
Large gap without hinge fracture.

Interactions between osteotome use and various baseline covariates on the incidence of LHF were analyzed within the PSM population (Table 3). The *p*-values for interactions involving large versus small wedge, correction amount, posterior slope change, and opening gap distance were all greater than 0.4. This indicated no significant difference in the effect of osteotome use on reducing LHF prevalence across these osteotomy subgroups. These results suggest that osteotome use’s benefits in decreasing the prevalence of LHF did not differ across the subgroups (Figure 3). Also, we did not find any difference between knees with hinge fractures when analyzing the other parameters as the different indications of osteotomy.

## Discussion

The aim of this article was to analyze on a scientific level the frequency of hinge fractures in tibial osteotomies when using osteotomes or oscillating saw, and to remind certain principles related to the surgeon’s experience. The discussion also approaches how this experience which can be summarized as a cognitive system dealing with unconnected medical devices (osteotome, saw, hammer) can be transform in a scientific approach with new tools such as vibration analysis coupled with artificial intelligence.

### Analysis of 2014 hinge fractures

No paper, to our knowledge, has been found on the advantage of osteotomes to prevent hinge fractures during OWHTO. The present study showed that using an osteotome reduced from 22% to 6% (*p* < 0.0001) the prevalence of hinge fracture and reduced the prevalence of clinically “important” hinge fracture from 6% to 2% (*p* < 0.0001). These data add new clinical findings regarding the eventual protective role of osteotome for the prevention of hinge fracture and consequences in high tibial osteotomy [27–32].

An osteotome is a very old tool in orthopedic surgery. There is no direct science about the prevention of osteotome on hinge fracture. However, making a less regular osteotomy line with several impacts of the osteotome intuitively explains the reduction in the frequency of fractures of the external hinge compared to a perfect osteotomy line with the oscillating saw. With a saw, the energy required to propagate a regular fracture is typically lower than that for an irregular fracture because the crack encounters fewer obstacles and follows a single cohesive plane, reducing the need for additional micro-cracks or material deformation around the crack path.

This study has limitations. Firstly, absence of prospective comparison between the use of different osteotomes and saws. Nonetheless, we believe this study includes the largest sample size reported in the literature on hinge fractures. Due to the limited number of hinge fracture cases in some subgroups, we had to consolidate multiple subgroups for interaction analyses. The study was, therefore, underpowered to evaluate outcomes within each subgroup. Similarly, the small number of clinically significant hinge fractures limited our ability to conduct stratified analyses based on specific indications for osteotomy [33–37] or associated techniques [38–40]. Thus, the study could only assess the overall relationship between osteotomy and clinically relevant hinge fractures requiring revision osteosynthesis.

### Epidemiology and diagnosis in the literature

In 2012, Takeuchi et al. [7] introduced the first classification for LHF, categorizing them into three distinct types: Type I fractures through the lateral cortex either proximally or at the proximal tibiofibular joint (TFJ); The “Type II” fractures extending distally to the TFJ, and “Type III” fractures involving the lateral tibial plateau. A biomechanical study by Kang et al. [40] found that “Type I LHF” had stability comparable to an open wedge high tibial osteotomy (OWHTO) with an intact hinge. Kang et al. [40], performed a recent biomechanical study, using a finite element model, and found that Takeuchi type I LHF exhibited stability similar to that of an OWHTO with an intact lateral hinge. In contrast, Takeuchi “types II and III” were considered as unstable. Clinical studies [41–46] align with this finding, with Nakamura et al. [47] reporting higher numbers of delayed union, correction’s loss or overcorrection in types II and III compared to type I LHF.

The incidence of LHFs has been ranges from 2% to 30%, primarily based on radiographic evaluations [48–52]. These fractures can occur during surgery or postoperative period, intraoperative rates around 18% and postoperative rates up to 14%. However, intraoperative fluoroscopy and standard postoperative radiographs may underestimate their occurrence. Lee et al. [52] demonstrated significantly higher LHF detection rates using CT scans compared to plain radiographs, with their study reporting the highest recognition incidence to date. Similarly, Kim et al.’s review [10] highlighted that CT scans improved LHF detection by 9.9% over radiographs alone, noting that up to 40% of LHFs might remain undetected with fluoroscopy or standard radiography.

#### Authors’ recommendation according to the senior surgeon experience

Independently of the various technical points concerning other aspects such as the radiological assessment [53–55], the tibial tuberosity [56–58], the senior author [59] with a personal experience of more than 2000 OWHTO with osteotome performed during four decades recommends several points to reduce the risk of hinge fractures: the postage stamp method, sensory feedback through sight and sound, tactile cues from the hammer, and vibrations from the osteotome.

*Postage Stamp Technique*: After performing an osteotomy, a 2.5 mm drill bit is directed obliquely from front to back through the posterior cortex [59], creating a pattern akin to a postage stamp. The final hole is drilled with a larger bit to create a slightly bigger opening at the hinge point, preventing splits at the osteotomy’s end, a practice dating back to 19th-century tibial bone grafting. Since the posterior tibia contains critical blood vessels and nerves, a retractor or “rugine” must protect this area during drilling. The bone osteotome is then positioned at the posterior osteotomy cortex to cut the cortex.

*Eye-Ear Coordination*: While cutting the lateral hinge cortex with a small bone chisel, the surgeon “listens” to the sound as the chisel contacts the external cortex, producing a dull tone [59, 60] signaling when to stop. A wide chisel is then used to open the osteotomy line. As the osteotomy deepens, bone stress concentrates at the apex. If the lateral cortex is not reached, more stress is exerted, raising fracture risk. Blunting the apex with a 5 mm drill hole reduces stress, easing the opening of the osteotomy.

*Tactile Feedback:* Surgeons use tactile sensations when guiding the osteotome, sensing bone resistance through their fingers. This tactile information helps gauge bone density and texture, allowing pressure adjustments for a precise, controlled cut. Variations in resistance alert the surgeon to shifts in bone hardness, guiding movement adjustments to avoid splintering [61].

*Vibrational Cues:* Striking the osteotome with a mallet or gentle pressure sends vibrations to the surgeon’s hands [59–61]. These cues vary based on bone elasticity and density, informing the surgeon of the cut’s depth and speed. This feedback enables pressure adjustments or more nuanced cuts.

*Elastic Resistance of the Bone:* Bone elasticity affects how it responds to the osteotome [61]. When force is applied, the bone compresses slightly and rebounds, signaling the bone’s tension. Softer, more elastic bones feel “forgiving,” while harder bones feel more resistant, allowing the surgeon to maintain precise control. The osteotomy is opened gradually, using bone elasticity by intermittently cleaving with a small chisel. Once open, a temporary wedge is placed before inserting the final wedge.

These techniques highlight the importance of sensory feedback, precision, and experience in managing hinge fractures effectively.

### From empirical feedback of the surgeon’s cognitive system to a decision-support system based on artificial intelligence

With experience, surgeons become finely attuned to feedback from different bone types, combining tactile and vibrational cues with the bone’s elasticity to shape it with precision. However, limitations in the human cognitive system – relying on a complex interplay of sensory systems like vision, proprioception, and motor control – make it challenging to transfer this nuanced expertise from one surgeon to another. Clustering different surgeon experience levels could benefit from artificial intelligence, as machine learning can help enhance novice surgeons’ performance.

Recent research works from our group has shown that some cognitive aspects in surgery can be measured. For example, a hammer “instrumented” with a piezoelectric force sensor located at its impacting face has been used for cementless total hip replacement with experimental insertion of cup and femoral stem [62–64]. This set-up allows to record the force as a function of time during an impact between the hammer and the ancillary (during a few milliseconds). The principle of the measurement is based on the energy transfer from the hammer to the ancillary-implant system, which bounces back and forth between the hammer and bone tissue. This phenomenon creates a resonance frequency that depends on the bone properties and that can be measured by the instrumented hammer. Based on this information, it was possible to assess: i) the stability and the acetabular cup, ii) the insertion endpoint of the femoral stem, and iii) femoral fractures in vitro, ex vivo, in silico and in anatomical subjects [64–66]. A similar approach was then used during osteotomy, where the implant-ancillary system is formally replaced by the osteotome. A proof of concept has been first evidenced in silico, and in vitro using bone mimicking phantoms and ex vivo [65–67]. It has then been validated in anatomical subjects in the context of rhinoplasty, pterogomaxillary disjunction, Summers osteotomy and, more recently, OWHTO [67–69]. This technique, referred to as Impact-Based Analysis Method (IBAM), can be used to track bone quality, osteotome progress, and detect potential fractures during surgery, offering real-time insights to enhance control and precision in various bone-shaping procedures. Our group is currently developing a decision-support system, and future clinical trials should be carried out to determine the performance of our approach.

## Conclusion

The osteotome may be an appropriate method for preventing hinge fractures following OWHTO. The European Society for Sports Traumatology, Knee Surgery, and Arthroscopy (ESSKA) acknowledges that the painful degenerative varus knee encompasses a wider range of indications than traditionally recognized [70–72]. To address this, ESSKA developed guidelines for treating such cases using joint-preserving methods. The finalized consensus offers comprehensive guidance on surgical techniques, rehabilitation strategies, and the management of complications related to knee osteotomies for degenerative varus knees.

## Data Availability

Data are available on request from the authors.
